# Public health determinants of screening accessibility, diagnostic and treatment timeliness, care adherence, and survival outcomes in breast cancer patients

**DOI:** 10.3389/fpubh.2026.1808014

**Published:** 2026-04-28

**Authors:** Meixia Zhang, Hui Pang, Beibei Cheng, Junfeng Deng, Wenjing Guo

**Affiliations:** 1 Medical College of Ordos Institute of Technology, Ordos, Inner Mongolia, China; 2 Department of Medical Oncology, Ordos Central Hospital, Ordos, Inner Mongolia, China

**Keywords:** breast cancer, care adherence, diagnostic delay, public health determinants, screening accessibility, treatment timeliness

## Abstract

**Background:**

Disparities in public health infrastructure, healthcare access, and system-level support contribute to delays in breast cancer screening, diagnosis, treatment initiation, and adherence to care, leading to advanced-stage presentation and poorer survival, particularly among rural and underserved populations.

**Aim:**

To evaluate the influence of individual, community, and health system level determinants on screening access, timeliness of diagnosis and treatment, adherence to therapy, and survival outcomes among women with breast cancer.

**Methods:**

This retrospective cohort study included 1,100 women diagnosed with primary breast cancer between January 2022 and December 2024. Data were obtained from hospital records, cancer registries, and administrative databases. Screening history, diagnostic and treatment intervals, treatment adherence, and survival were assessed. Multivariable logistic regression identified determinants of screening access, diagnostic and treatment delays, and treatment non-completion, while Cox proportional hazards models evaluated survival after adjustment for confounders.

**Results:**

Over one-third of patients had never undergone prior screening, and 25% traveled more than 25 km to access services, indicating that geographic and access barriers may contribute to delayed diagnosis and poorer survival. Diagnostic delays exceeding 60 days occurred in nearly one-third of patients, and one-quarter experienced treatment initiation delays. Overall treatment adherence was 72.1%, with lower adherence among uninsured patients. Integrated pathway analysis identified a low-access group (28.3%) characterized by absent screening, who had prolonged delays at each stage of care and a 32.5% mortality at follow-up, and reduced survival, whereas the high-access group demonstrated >90% survival at follow-up. Geographic, financial, and system-level barriers were independently associated with delays, treatment non-completion, and poorer survival. In contrast, care at tertiary centers and patient navigation support were significantly associated with improved timeliness, adherence, and survival outcomes.

**Conclusion:**

Social and system-level determinants exert cumulative effects across the breast cancer care continuum. Strengthening healthcare access, expanding patient navigation, and enhancing system coordination may reduce delays, improve adherence, and improve survival.

## 1 Introduction

Breast cancer is an issue of high prevalence in women all over the world, representing one of the leading causes of cancer-related morbidity and mortality among females globally. It is also major challenge to the overall health of people because of the existing inequality in breast cancer prevention, early diagnosis, and prognosis. Although there have been improvements in screening technologies and treatment modalities, disparities in access to breast cancer screening services remain substantial across different populations and regions, particularly in low- and middle-income countries and underserved communities. The social and structural factors, including income, education, health insurance cover, geography, and capacity of the healthcare system, have a substantial impact on the participation in screening programs and result in unequal opportunities in the early detection. These disparities are increasingly recognized as key drivers of cancer inequities and are central to public health research and policy. These differences in access to screening also aid in the delayed diagnosis, more advanced disease manifestation, and poorer prognostic outcomes, highlighting the critical role of population health determinants in shaping breast cancer outcomes across the care continuum (1, 2).

The correct sequence of actions leading into the diagnostic confirmation of the abnormal screening results and the start of treatment is also a paramount determinant of breast cancer outcome. Access to healthcare, especially specialization and transportation facilities, at the community level, has been found to play a major role in the determination of the diagnostic and treatment process. Timely transitions between each stage of care from screening to diagnosis and from diagnosis to treatment are essential components of an effective cancer care continuum. Stalling points in the care continuum usually come about as a result of fragmented referral procedures, lack of oncology services as well as disparities in healthcare resources allocation. Such barriers of the system have a disproportionate impact on underserved and rural populations, which leads to a long term delay in initiating treatment and the further development of the disease (3, 4). The patient level, provider-level, and health system level factors increase delays in the diagnosis and management of patients in low- and middle-income settings. Lack of awareness about breast cancer symptoms, sociocultural beliefs, economic factors, and misdiagnosis at the primary care level are some of the factors that lead to late presentation and extended time periods of diagnosis. In addition, limited access to diagnostic technologies, inadequate referral pathways, and shortages of trained oncology professionals further exacerbate these delays. These problems are exacerbated by inefficiencies in health systems such as referral delays and insufficient diagnostic capacity, which make it difficult to manage them in time. Specifically, these delays are especially harmful because they are closely connected to the diagnosis of disease at its advanced stages and lower chances of survival (5, 6).

In addition to access and timeliness, compliance with screening, diagnostic follow-up and treatment protocols is an essential determinant of the outcome of breast cancer. Continuity of care across the cancer care continuum is essential for achieving optimal clinical outcomes and improving survival. Vulnerable populations, including disabled women and socioeconomically disadvantaged groups, face structural and communication barriers that restrict their participation in screening services and continuity of care. Moreover, unprecedented public health outbreaks, like the COVID-19 pandemic, have brought to the limelight vulnerabilities in healthcare systems, disrupting screening services, creating delays in the diagnostic process, limiting treatment compliance, and exposing people to telemedicine. These disruptions have further widened existing disparities in cancer care delivery and outcomes (7, 8).

Even in developed health care systems, the difference in the timeliness of diagnostic and treatment remains, especially in patients with metastatic disease and with socially disadvantaged backgrounds. It has been observed that the delay of treatment has been linked to a worse outcome of survival and higher risk of death and thus delivery of care must be fair and timely. Psychosocial factors such as fear, low health literacy, and cultural perceptions toward cancer screening also influence healthcare-seeking behavior and adherence to recommended care pathways. Attitudinal fears, health literacy, and sociocultural feelings toward screening, e.g., as it is the case with the Middle Eastern populations, contribute to the increased screening uptake and adherence to it, which validates the necessity of context-specific interventions at the level of the public health. All these complex determinants must be addressed to enhance the global survival rates and decrease inequalities in breast cancer (9, 10).

Even though there is a large amount of literature on breast cancer, most studies have done individual studies on screening access, diagnostic delay, timeliness of treatment, adherence and survival outcomes. Some minimal knowledge on the interaction of these factors within the continuum of care is left. Also, the majority of the current evidence is on individual or clinical determinants on an individual level, and there is relatively little on large-scale determinants of the socioeconomic inequalities, healthcare system capacity, and barriers in the structure. This piece meal approach reduces the possibility of comprehending cumulative disadvantages along the breast cancer care pathway. There is limited evidence especially in low- and middle-income countries and other sociocultural contexts that also highlights the barriers on the screening-to-treatment continuum.

Thus, the current study focuses on assessing the determinants of individual, community, and health system levels regarding breast cancer care on the entire care continuum, such as access to screening, timeliness of diagnostic and treatment, care adherence, and survival outcome. Moreover, this research aims to investigate the aggregate effect of waiting time and non-compliance on survival rates and determine the role of interaction effects between social factors of determinants of public health and contribution to inequalities in breast cancer treatment. Through a continuum-based approach, the proposed study will seek to establish evidence in order to inform interventions and policies related to health to reduce disparities and enhance outcomes in breast cancer patients.

## 2 Methodology

### 2.1 Study design and duration

This study was a retrospective multicenter cohort study that was done in 6 health institutions (3 tertiary cancer centers, 2 general hospitals and 1 district-level facility) located in Ordos, Inner Mongolia, China. The populations of both urban and rural areas were included in a specific regional cancer care network. Institutional cancer registries were used to identify cases, and cross-linked with hospital electronic medical records. Deterministic matches between patient-level data in: hospital EMRs, cancer registry databases, administrative health records were made by unique patient identifiers. The period of the study was January 2022 to December 2024, which was long enough to follow up and measure the treatment adherence and survival rates and also represent the current trends in the healthcare delivery process.

### 2.2 Setting and data sources of the study

The research environment involved a system of tertiary and secondary care providers of integrated breast cancer screening, diagnostic, and oncology services. Several sources of information were used, such as hospital EMRs, cancer registries, screening program databases and administrative health records. By implicating such integrated sources of data, patient-level data, such as demographic attributes, screening history, diagnosis timeline, treatment courses, adherence metrics, and clinical outcomes were comprehensively captured. Moreover, geospatial and administrative data associated with the residential location of patients were also used to obtain the indicators of healthcare access at the community level, as it has been previously applied in the research on healthcare access.

### 2.3 Population and sample size of the study

**Figure 1:**
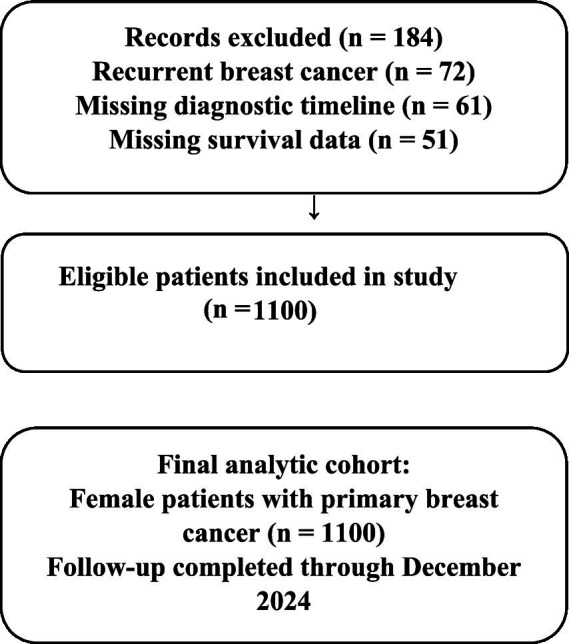
Participants flow diagram.

A total of 1,284 patient records were initially identified from institutional cancer registries and hospital databases. After applying eligibility criteria, 184 cases were excluded due to recurrent breast cancer (*n* = 72), missing diagnostic timeline data (*n* = 61), or incomplete survival data (*n* = 51), resulting in a final analytic cohort of 1,100 female patients with histologically confirmed primary breast cancer diagnosed or treated during the study period as shown in Figure 1. Eligible participants were adults with at least one recorded encounter with diagnostic or screening services and complete documentation of key exposure and outcome variables. Missing data were handled according to the extent of incompleteness which minimizes bias in multivariable analyses and ensures robust estimates for determinants of care. Complete-case analysis was applied for variables with less than 5% missingness, while multiple imputation using chained equations was used for variables with 5%–15% missing data. Follow-up was ascertained through linkage with cancer registry and hospital records, with vital status determined up to December 2024. The sample size was adequate to support multivariable analyses of access, timeliness, adherence, and survival outcomes, including subgroup analyses.

### 2.4 Operational definitions

#### 2.4.1 Screening exposure

Exposure to screening has been grouped into three, namely, regular screening, which refers to at least one screening episode within 24 months of the diagnosis; irregular screening, which refers to screening done more than 24 months before diagnosis; and no screening referred to lack of any recorded screening before the diagnosis.

#### 2.4.2 Diagnostic delay

This was considered as the duration of time more than 60 days between first clinical manifestation or abnormal screening finding and histopathologic determination of breast cancer.

#### 2.4.3 Treatment delay

Treatment delay was considered as more than 30 days between confirmed diagnosis to starting the first definitive treatment be it surgery, chemotherapy, radiotherapy or hormonal therapy.

#### 2.4.4 Care adherence

The operationalization of the care adherence was the completion of the treatment. Adherence to treatment was determined as completion, which was considered to be at least 90% of the prescribed regimen, partial adherence which was considered completion of 50%–89%, non-adherence which was considered less than 50% completion or discontinuation of treatment.

#### 2.4.5 Patient navigation exposure

Exposure to patient navigation was identified as recorded structured assistance in the coordination of appointments, promotion of referrals, and assistance with the maintenance of follow-up care during the journey.

#### 2.4.6 Community access index

Community access index was created as composite index that included density of healthcare facilities, travel distance to the healthcare facilities and the accessibility of screening programs. This index was divided into tertiles that were defined as high, moderate and low access levels.

#### 2.4.7 Deprivation index

The deprivation index was calculated based on area level socioeconomic measures, such as income level, educational attainment and employment status, which describe the relative socioeconomic disadvantage of residential areas of patients.

#### 2.4.8 Facility type

The type of facility at diagnosis was defined into three categories; tertiary (specialized oncology facilities that offer complete cancer care facilities), secondary (general hospitals offering diagnostic and curative services), and primary-level facilities (district healthcare centers with a limited diagnostic capacity).

### 2.5 Public health determinants and exposure variables

Conceptualization of the public health determinants was in individual, community, and health system levels. The variables of individual level were age of diagnosis, educational level, socioeconomic measurements, insurance or financial assistance level, comorbidities and reported health-seeking behavior. The community level determinants included the healthcare facility density, the distance to screening and treatment facilities, urban–rural residential place, and the deprivation index in neighborhoods. The determinants of the health system were availability of systemized screening programs, referral pathways, patient navigation, diagnostic capabilities and treatment service availability. These determinants were operationalized based on composite indices and categorical measurements based on previous empirical models that assessed healthcare accessibility and system performance.

### 2.6 Evaluation of screening accessibility

Screening access was assessed through analysis of the past experience of breast cancer screening programs and the last time accessing screening programs in relation to getting a diagnosis and reported barriers to screening. The indicators of access covered both the physical access, including distance and the availability of the services, as well as functional access, including the availability of appointments and referral mechanisms because physical and functional access barriers are known predictors of late-stage presentation. The patients were grouped based on the screening habits of regular screening, delayed screening, and no screening before diagnosis. This analysis has allowed the consideration of the impact of the structural and contextual factors on early detection.

### 2.7 Diagnostic timeliness

Diagnostic timeliness was set as time of the initial presentation of symptoms or abnormal screening results to breast related symptom or unusual screening result and the time of histopathological diagnosis of breast cancer. The duration of time was computed by using the recorded dates of first healthcare contact, diagnostic radiology, biopsy, and definite diagnosis. Clinically meaningful thresholds that were used to operationalize delayed diagnosis were operationalized using established standards of oncology care. Diagnostic delay determinants were determined as dependent on the individual, community, and system factors, such as the efficiency of the referral and diagnostic service capacity.

### 2.8 Pathways and timeliness of treatment

The timeliness of treatment was measured as the duration between first definitive treatment, which could be surgery, chemotherapy or radiotherapy or hormonal treatment after being diagnosed. Other intervals like time to complete multimodal therapy were also assessed. The pathways of treatment were divided into compliance with the prescribed clinical schedules to evaluate how delays in initiation affect completion of therapy and survival. The impact of access to healthcare, patient navigation services, and institutional capacity on initiation and continuation of treatment was tested to determine the delays within the system.

### 2.9 Care adherence

The care adherence was assessed by the completion of the recommended treatment regimes, attendance of scheduled oncology appointments, and adherence to prescribed treatments. The indicators of adherence were treatment discontinuation, absenteeism, and non-adherence to the care plans on the basis of the guidelines. The association between patient navigation service, socio-economic barriers and health system support mechanisms on promoting or discouraging adherence was examined. This component indicated the interplay between structure level healthcare determinants and patient level behaviors.

### 2.10 Survival outcomes

The outcome of survival was measured in terms of overall survival and disease-specific survival, which were estimated between the date of diagnosis up to the date of death or the date of the last follow-up. The survival analyses were able to consider stage at diagnosis, timeliness of treatment, pattern of adherence, and access-based determinants. Patients that had not been documented as dead within the study time were censored on the last known date. This design allowed assessing the relationship between differences in access and care processes and the difference in survival.

### 2.11 Statistical analysis

Patient characteristics, access indicators, care timelines, and outcome measures were summarized using descriptive statistics. Multivariable regression analyses were performed to identify independent predictors of screening access, diagnostic delay, treatment delay, and care adherence. Logistic regression models were used to assess binary outcomes, including absence of pre-screening history, delayed diagnosis, delayed treatment, and failure to receive treatment. The association between public health determinants and survival outcomes was evaluated using Cox proportional hazards models, with overall survival as the primary endpoint. All models were adjusted for relevant confounders selected based on prior literature and clinical relevance. Interaction terms were included to assess synergistic effects between community-level access and health system factors.

Multicollinearity was assessed using variance inflation factors (VIF), with values below 5 indicating no significant collinearity. Robust standard errors were applied to account for potential clustering across healthcare centers. Model diagnostics were systematically evaluated. The Hosmer-Lemeshow test was used to assess the goodness-of-fit of logistic regression models, while the proportional hazards assumption of Cox models was examined using Schoenfeld residuals. Survival distributions were estimated using the Kaplan–Meier method and compared using log-rank tests. Statistical significance was defined using conventional thresholds.

### 2.12 Ethical considerations

This research was approved by the Ethics Committee of Medical College of Ordos Institute of Technology, Approval Number: EYY2025000003. Data collection was done after getting the relevant institutional review boards to provide ethical approval. The treatment of the patient records was de-identified to ensure patient confidentiality and keep the data securely. As the study is retrospective, the informed consent was not needed, as per the national and institutional ethical standards.

## 3 Results

### 3.1 Characteristics of the population of the study

1,100 breast cancer patients passed the eligibility criteria and were used in the final analysis. The cohort was a heterogeneous group in terms of age, socioeconomic status, geographic location and health system exposure. A majority of the patients were diagnosed in the process of active participation in the public or a combination of the public and privatized medical services. Prior screening exposure, availability of structured referral channels and the availability of patient navigation support were found to vary. The characteristics of tumors included early-stage disease diagnosed by the screen to advanced symptomatic cases, which depict different points of care continuum entry.

Table 1 displays baseline characteristics in individual, community, and health system levels. Patients in younger age groups and those living in urban or peri-urban areas were more often associated with organized screening services, and older patients and those in underserved groups of the population were more associated with symptomatic presentation. Disagreements were also identified in the insurance cover, education level, comorbidity burden and accessibility to oncology facilities.

**Table 1:** Baseline characteristics of the study population (*N* = 1,100).

Variable	Category	*n* (%)
Age group (years)	<40	198 (18.0)
	40–49	312 (28.4)
	50–59	341 (31.0)
	≥60	249 (22.6)
Residence	Urban	472 (42.9)
	Peri-urban	348 (31.6)
	Rural	280 (25.5)
Education level	No formal/primary	301 (27.4)
	Secondary	417 (37.9)
	Tertiary	382 (34.7)
Health insurance	Full	389 (35.4)
	Partial	462 (42.0)
	None	249 (22.6)
Comorbidities	None	514 (46.7)
	One	371 (33.7)
	≥Two	215 (19.6)
Facility type at diagnosis	Tertiary cancer center	483 (43.9)
	General hospital	417 (37.9)
	District facility	200 (18.2)
Patient navigation exposure	Yes	468 (42.5)
	No	632 (57.5)

Percentages are not necessarily equal to 100 because they were rounded off. Patient navigation can be defined as an organized support of patient appointments, referrals, and treatment coordination. This heterogeneity suggests that rural and older patients may be at higher risk of delayed detection and advanced-stage disease.

### 3.2 Screening accessibility and mode of presentation

The access to screening differed greatly among the community access strata. The patients who lived in a region with a greater concentration of healthcare facilities and regular screening campaigns had a higher probability of receiving mammography or clinical examination of the breast before being diagnosed. By contrast, patients in the lower-access setting often came into care when the symptoms became apparent. There was also the influence of education level, insurance cover and distance to screening facilities in prior screening exposure (Table 2).

**Table 2:** Screening accessibility and presentation patterns.

Variable	Category	*n* (%)
Prior screening history	Regular screening	398 (36.2)
	Irregular screening	317 (28.8)
	No prior screening	385 (35.0)
Mode of presentation	Screen-detected	361 (32.8)
	Symptomatic	739 (67.2)
Travel distance to screening	≤10 km	422 (38.4)
	11–25 km	387 (35.2)
	>25 km	291 (26.5)
Community access index	High	366 (33.3)
	Moderate	421 (38.3)
	Low	313 (28.5)

Cases that were identified during screens were more common in patients who lived nearer to screening facilities as well as in communities with greater access to facilities whereas symptomatic presentation was predominant in localities with limited diagnostic services. Patients with no prior screening were more likely to present symptomatically, highlighting the impact of limited access and education on early detection.

### 3.3 Diagnostic timeliness

Diagnostic intervals between subgroups of patients varied notably. Patients that had undergone previous screening or direct referrals to tertiary or specialized centers also had a less protracted diagnosis pathway, but protracted time was noted in patients who were initially assessed at the primary or lower-level care centers and had undergone multiple referrals. These wastes could be mainly explained by the limitations of the health system, such as low capacity to diagnose, short imaging access, long biopsy turnaround time, and poor navigation of the patients (Table 3).

**Table 3:** Diagnostic time intervals from first presentation to histological confirmation.

Diagnostic interval	Category	*n* (%)
≤30 days	—	412 (37.5)
31–60 days	—	358 (32.5)
61–90 days	—	211 (19.2)
>90 days	—	119 (10.8)
Initial point of care	Tertiary	483 (43.9)
	Secondary	379 (34.5)
	Primary	238 (21.6)
Multiple referrals	Yes	327 (29.7)
	No	773 (70.3)

Prolonged diagnostic intervals were concentrated in patients referred from primary care, indicating that referral efficiency directly influences stage at diagnosis and survival.

### 3.4 Treatment timeliness and care pathways

Delays in starting treatment based on confirmed diagnosis depended on the type of treatment, type of facility, and the assistance of a navigator. Patients that were treated in specialized oncology facilities and those served by the navigation services typically started their treatment earlier. The delays were also higher when the use of multimodal therapy was necessary or when there was cross-facility coordination in the treatment planning (Table 4).

**Table 4:** Time from diagnosis to initiation of first definitive treatment.

Time to treatment initiation	Category	*n* (%)
≤30 days	—	446 (40.5)
31–60 days	—	381 (34.6)
61–90 days	—	189 (17.2)
>90 days	—	84 (7.6)
Primary treatment modality	Surgery first	504 (45.8)
	Neoadjuvant therapy	379 (34.5)
	Non-surgical systemic	217 (19.7)
Navigation support	Present	468 (42.5)
	Absent	632 (57.5)

Timely initiation of treatment was more effective in patients who received care in integrated care pathways and longer timelines were seen when inter-service coordination had to be made. Delays in treatment initiation were associated with lower adherence and could contribute to poorer survival.

### 3.5 Care adherence and treatment completion

There was a disparity in compliance to suggested treatment regimens depending on the socioeconomic and health system settings. Patients who had the support of the navigation had better completion rates of the planned therapies and follow-up visits. Obstacles to compliance comprised treatment related toxicity, economic problems, travel burden, and service disruptions (Table 5).

**Table 5:** Care adherence indicators.

Adherence indicator	Category	*n* (%)
Completion of planned treatment	Yes	793 (72.1)
	Partial	207 (18.8)
	Discontinued	100 (9.1)
Missed ≥1 oncology appointment	Yes	346 (31.5)
	No	754 (68.5)
Treatment interruptions	Yes	281 (25.5)
	No	819 (74.5)
Financial barrier reported	Yes	318 (28.9)
	No	782 (71.1)

It was found that patients with a lower adherence pattern were more frequently affected by the barriers of money and distance, which underlines the importance of the support mechanisms on the system level. Patients facing financial and geographic barriers were less likely to complete treatment, demonstrating the interplay between socioeconomic status and care adherence.

### 3.6 Survival outcomes

The survival results differed at the end of the follow-up period depending on the stage of diagnosis, timeliness of the treatment, and adherence patterns. The patients who had been diagnosed at an earlier stage and those who had undergone suggested courses of treatment had longer survival rates. Late diagnosis and treatment was linked with increased mortality within the period of observation (Table 6).

**Table 6:** Survival outcomes by key care indicators.

Outcome	Category	*n* (%)
Vital status	Alive	892 (81.1)
	Deceased	208 (18.9)
Stage at diagnosis	Early (I-II)	514 (46.7)
	Advanced (III-IV)	586 (53.3)
Treatment completion	Completed	793 (72.1)
	Not completed	307 (27.9)
Median follow-up duration	—	~30 months

Cumulative effects demonstrating that early screening, timely diagnosis, and treatment adherence collectively improve survival outcomes.

**Figure 2:**
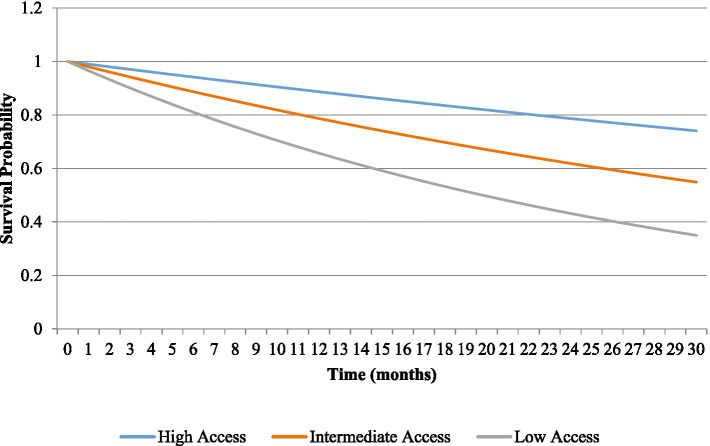
Kaplan–Meier survival curves by care continuum pathway.

The survival analysis using Kaplan–Meier showed that there was a significant difference in survival probability among groups in the care continuum. The patients in the high-access pathway were the most likely to survive through the follow up period, with the patients in the low-access pathway having very low chances of survival. The mid-access population showed the survival results that exist between these two extremes. The differences proved to be statistically significant according to log-rank test (Figure 2).

### 3.7 Integrated analysis across the care continuum

Gradient patterns became different when the determinants of population health were measured together throughout the breast cancer care continuum through the access of screening, the timeliness of the diagnosis, initiation of the treatment, adherence to care, and the survival rates as shown in Table 7. Latent modeling was not used to classify the patients into care continuum profiles but instead it was based on predefined criteria. Regular screening, the diagnostic interval, and the initiation of treatment within ≤30 days were determined to be the high-access pathway. The intermediate-access route involved patients whose patterns of screening, moderate delays in either diagnosis or treatment entry (61–90 days and 31–60 days) were mixed. The low-access pathway entailed patients that had never been screened previously, long diagnostic periods (more than 90 days), and late onset of treatment (more than 60 days).

**Table 7:** Integrated care continuum trajectories and outcomes.

Care continuum profile	Screening access	Diagnostic interval	Treatment initiation	Treatment completion	Vital status at follow-up
High-access pathway (*n* = 368)	Regular	≤60 days	≤30 days	86.4%	91.0% alive
Intermediate-access pathway (*n* = 421)	Irregular	61–90 days	31–60 days	73.2%	82.7% alive
Low-access pathway (*n* = 311)	None	>90 days	>60 days	54.0%	67.5% alive

These created categories showed similar associations in various care stages. The patients with a low-access pathway had higher chances of cumulative disadvantages such as late access to care, a lengthy duration of diagnosis, lower chances of treatment, and poor outcomes of survival at follow-up. On the contrary, the people of the high-access pathway had more preferable trajectories, where they had correct care delivery, had better adherence to care, and better survival. These results underscore the cumulative and interactive nature of the determinants of public health and the systematic level throughout the continuum, meaning that constraints at an earlier stage of the continuum are carried on to the next stage of care, ultimately affecting the outcomes of survival.

Low-access patients showed compounded disadvantages at each care stage, resulting in markedly lower survival rates.

### 3.8 Integrated multivariable analysis across the breast cancer care continuum

Geographic and socioeconomic determinants proved to be associated with various stages of care. Living in the rural area, long travel distance to health care centers, and not having insurance cover were linked with low screening rates and protracted diagnosis and treatment processes, which are further translated to less treatment adherence and increased mortality along the follow-up. The protective associations between the care continuum and the health system-level facilitators were shown to be protective. Treatment access to a tertiary cancer center and patient navigation support were linked to reduced diagnostic and treatment time, greater treatment adherence, and better patient survival, which suggests that coordinated and increased access to care can reduce cumulative barriers to access.

Table 8 shows the adjusted relationships of the main determinants of public health and the outcomes at consecutive stages of the breast cancer care continuum, such as access to screening, timeliness of diagnosis, treatment start, treatment adherence, and survival. Every column is an independent multivariable model, and one can compare the effect of the same determinants on different stages of care instead of the standalone results.

**Table 8:** Adjusted associations between public health determinants and outcomes across the breast cancer care continuum (*N* = 1,100).

Determinant	No prior screening OR (95% CI)	Prolonged diagnosis OR (95% CI)	Delayed treatment OR (95% CI)	Non-completion OR (95% CI)	Mortality HR (95% CI)
Rural residence	1.48 (1.16–1.90)	1.32 (1.02–1.71)	1.27 (0.98–1.64)	1.41 (1.08–1.84)	1.33 (1.01–1.76)
Travel distance >25 km	1.63 (1.25–2.13)	1.46 (1.12–1.91)	1.39 (1.05–1.83)	1.51 (1.14–2.01)	1.42 (1.07–1.89)
No insurance	1.71 (1.31–2.24)	1.29 (1.00–1.66)	1.44 (1.09–1.90)	1.88 (1.42–2.49)	1.37 (1.02–1.85)
No navigation support	—	1.42 (1.10–1.83)	1.61 (1.24–2.09)	1.74 (1.31–2.31)	1.49 (1.11–2.00)
Tertiary cancer center	0.62 (0.48–0.80)	0.69 (0.54–0.88)	0.71 (0.56–0.91)	0.66 (0.51–0.86)	0.73 (0.55–0.96)
Advanced stage at diagnosis	—	—	1.34 (1.05–1.72)	1.27 (0.99–1.63)	2.14 (1.62–2.83)

The odds ratios (ORs) are provided on the absence of previous screening, long diagnostic durations, late treatment, and non-treatment. Mortality has hazard ratios (HRs). The columns indicate individual multivariate models that have been corrected by age at diagnosis, educational level, comorbidity burden and year of diagnosis. Dashes (−) mean that a variable is not present in a model since it is conceptually and/or temporally irrelevant. Rural residence, long travel distance, and lack of insurance independently increased the odds of delayed care and mortality, whereas patient navigation and tertiary care access mitigated these risks.

## 4 Discussion

### 4.1 Population characteristics and structural inequities

The sample of 1,100 breast cancer patients used in this study had a heterogeneous composition of age, comorbidity, and socioeconomic status as well as geographically dispersed representation. Individuals living in urban or peri-urban regions and younger patients were more likely to attend organized screening programs and older patients and those living in rural and underserved population presented with symptomatic disease. This observation is consistent with earlier literature indicating that the age, urban city, and socioeconomic status have an interaction that determines access to healthcare and the use of preventive care (11, 12). The burden of comorbidity also seemed to affect the course of treatment: multiply morbid patients were likely to experience a delay in the referral pathway to treatment, pathway to diagnosis, and entry into treatment, as previously observed in geriatric settings, where comorbidity is an issue complicating the implementation of guidelines (13). The difference in insurance coverage and education level also implies the presence of systemic disparities that contribute to delays and narrow the range of treatment options, which underlines the significance of structural interventions to address underserved groups.

### 4.2 Screening accessibility and mode of presentation

The exposure to screening was closely related to the availability of the healthcare facility in the communities, screening history, education and insurance cover. High-access areas tended to provide mammography or clinical breast examination prior to symptom formation and low-access communities tended to receive symptomatic presentation. The results are consistent with the existing literature that indicates that geographic barriers, low health literacy, and insufficient structured screening programs are involved in late diagnosis of vulnerable groups (14, 15). It is worth mentioning that partial engagement with screening may confer survival benefits, emphasizing the need for community outreach (16). Sociocultural influences such as awareness, stigma, and attitude toward breast cancer can also further mediate the screening adoption as was seen in the South Asian population (17).

### 4.3 Diagnostic timeliness

The time interval between diagnoses differed significantly among groups of patients. Patients who were previously screened or directly referred to tertiary centers recorded a lower interval between the initial presentation and histological confirmation, while patients who were initially examined at the primary care centers or needed sequential referrals had longer delays. These delays were associated with system level constraints (like limited diagnostic capacity, imaging bottleneck, and biopsy turnaround time) which has also been found in low- and middle-income countries (18, 19). Patients who commuted more than 25 km to diagnostic facilities also had longer diagnostic intervals, which supports the finding that travel burden is linked to advanced stage of presentation (20, 21). Our statistics reduce time-to-diagnosis, stage progression, and mortality, simplify referral, and provide equal access to the imaging and pathology services to minimize delays and provide patients with improved pathways.

### 4.4 Treatment timeliness and care pathways

Facility type, support in form of navigation, and modality of care played a role in influencing treatment initiation. Patients that had been treated in tertiary cancer centers or with the help of navigators initiated treatment earlier, whereas those with multimodal treatment or cross-facility coordination experienced a delay. These results support the previous data that integrated care pathways and multidisciplinary coordination contribute to the better efficiency of treatment and decreased time-to-treatment factors (22, 23). Notably, the information also demonstrates that patient navigation can improve outcomes even in resource-constrained settings present at the systemic level, which will help to deliver care in time even in the resource-constrained environment (24, 25). The combination of treatment modality and delay of care supports the importance of individual care planning, particularly in patients with the necessity to receive the neoadjuvant therapy or sophisticated surgical operations.

### 4.5 Care adherence and treatment completion

Both system-level and patient-level factors affected treatment adherence to prescribed treatments. Patients who received aid in navigation recorded better completion rates, reduced missed appointments, and interruption of treatments, and financial limitations, travel burden, and toxic complications were associated with a serious impediment in low adherence groups. This result is evidenced by the fact that patient navigation enhances continuity and treatment adherence in various population groups (26, 27). It is important to note that social economic and geographic factors interplayed with health systems burdens to cause different adherence patterns and that any intervention aimed at increasing compliance should be multi-dimensional, involving economic support, travel logistics, psychosocial support, and education (25).

### 4.6 Survival outcomes

Stage of diagnosis, timeliness in treatment, and compliance to treatment was closely related to survival outcomes. The patients diagnosed at the early stages and undergoing prescribed treatment showed better survival, whereas late-stage diagnosis and failure to receive the treatment were the factors that linked with less survival chances. Kaplan–Meier analysis found associative differences in high-, intermediate-, and low-access pathways, where the effect of barriers along the care pathway is cumulative (28, 29). These results highlight the interdependence of early diagnosis, timely therapy, and treatment compliance as the determinants of survival, as systematic reviews indicate that guideline-concordant care lowers the level of mortality in a wide range of settings (30, 31). Furthermore, the difference in survival rates between low-access patients highlights the importance of active initiatives in the field of population health in order to diminish outcome inequality.

### 4.7 Integrated care continuum and cumulative disadvantage

Combined care pathway analysis showed that cumulative inequalities developed as disadvantages at earlier stages of the continuum were transmitted to later ones. The low-access pathway patients were found to have delayed screening and long diagnostic time, late onset of treatment, low adherence rate, and low survival, compared with high-access pathway patients who recorded good outcomes all through. Such trend underlines the idea of cumulative disadvantage, in which inequities at early stages are carried down to the realm of diagnosis, treatment, and survival (21, 32). The results highlight the significance of system-wide and integrated interventions that tackle obstacles at multiple stages at a time as opposed to addressing the obstacles at distinct stages of care individually (33). The multi-level strategies such as community outreach, capacity building in primary care, and strong navigation programs could help avoid the multiplication of initial inequities and achieve equity throughout the care continuum.

### 4.8 Multivariable determinants across the care continuum

The multivariable analysis showed that geographic, socioeconomic, and system-level determinants were differentially related to the outcomes at both stages. Living in the rural area, increased travel distance and absence of insurance were always linked with low screening rates, longer diagnostics and treatment periods, low treatment adherence, and an increased mortality rate. In contrast, the shortest diagnostic and treatment time, adherence, and survival were related to the availability of tertiary cancer centers and navigation assistance (33–35). These observations imply that the most effective way of reducing the cumulative disparities in breast cancer care could be interventions that focus on multiple determinants at once, instead of individual ones. Further, the presence of advanced diagnosis at the time of diagnosis was a powerful predictor of mortality, which demonstrates that the early detection and system-level provision are crucial to the development of survival outcomes (18, 20).

#### 4.8.1 Strengths of the study

The current research is an analytical study that involves a detailed evaluation of breast cancer care throughout the continuum, incorporating screening, diagnosis, treatment, adherence and survival into a single platform of analysis. The implementation of a very large multi-centric cohort of 1,100 patients increases the external validity of the results in a variety of healthcare contexts. The integration of individual-, community-, and system-level determinants provided the opportunity to evaluate in detail structural barriers and contextual barriers to care. Integrated pathway analysis and multivariable models used facilitated the discovery of cumulative effects at various stages of care, providing policy-specific actions to the public health and health system planning.

#### 4.8.2 Limitations of the study

The retrospective design did not permit causal relationships to be established and depended upon previous medical records that were available and accurate. Some variables, e.g., patient-reported barrier, psychosocial factor and informal care-seeking behavior were unavailable or under-reported. Differences in records among healthcare centers could have affected the measurement process of diagnostic and treatment intervals and adherence measures. Also, the study by definition was restricted to survival follow up during this period which is possibly not representative of long-term outcomes after December 2024.

#### 4.8.3 Future recommendations

Prospective designs should be used in future research to understand the dynamic variations in access, adherence, and outcomes with time. Inclusion of qualitative assessment may help to gain better understanding of patient-level barriers and decision-making. The growth and assessment of the patient navigation programs, as well as the decentralized diagnostic services, could enhance timely access to the care and adherence. Policymakers ought to target the decrease in the geographic and financial barriers and greater coordination among referrals to streamline the care pathways. It is suggested to have the long-term follow up studies to evaluate the long term survival effects and the system level interventions.

## 5 Conclusion

The impact of the determinants of health on the population on the prevention of breast cancer is cumulative and intertwined on every level of the breast cancer care life cycle. Delayed screening, diagnosis, treatment, less adherence, and poor survival were always linked to structural barriers, such as rural residence, prolonged travel distance, the absence of insurance, and poor patient navigation. On the other hand, treatment provided at the tertiary cancer facilities and with the help of the navigation, services were linked to the more timely care, better treatment adherence, and better survival rates. These determinants need to be addressed using integrated approaches to public health to achieve equity, optimal care delivery, and survival among breast cancer patients.

## Data Availability

The raw data supporting the conclusions of this article will be made available by the authors, without undue reservation.
